# Detecting Nanotopography Induced Changes in Cell Migration Directions Using Oxygen Sensors

**DOI:** 10.3390/bios14080389

**Published:** 2024-08-12

**Authors:** Muting Wang, Stella W. Pang

**Affiliations:** Department of Electrical Engineering, Centre for Biosystems, Neuroscience, and Nanotechnology, City University of Hong Kong, Hong Kong, China

**Keywords:** directed cell migration, cell migration directional change, O_2_ detection of single cells, O_2_ biosensors, fluorescence of PtOEPK dye, mitochondria

## Abstract

This study investigates the oxygen (O_2_) consumption of single cells during changes in their migration direction. This is the first integration of nanotopographies with an O_2_ biosensor in a platform, allowing the real-time monitoring of O_2_ consumption in cells and the ability to distinguish cells migrating in the same direction from those migrating in the opposite direction. Advanced nanofabrication technologies were used to pattern nanoholes or nanopillars on grating ridges, and their effects were evaluated using fluorescence microscopy, cell migration assays, and O_2_ consumption analysis. The results revealed that cells on the nanopillars over grating ridges exhibited an enhanced migration motility and more frequent directional changes. Additionally, these cells showed an increased number of protrusions and filopodia with denser F-actin areas and an increased number of dotted F-actin structures around the nanopillars. Dynamic metabolic responses were also evident, as indicated by the fluorescence intensity peaks of platinum octaethylporphyrin ketone dye, reflecting an increased O_2_ consumption and higher mitochondria activities, due to the higher energy required in response to directional changes. The study emphasizes the complex interplay between O_2_ consumption and cell migration directional changes, providing insights into biomaterial science and regenerative medicine. It suggests innovative designs for biomaterials that guide cell migration and metabolism, advocating nanoengineered platforms to harness the intricate relationships between cells and their microenvironments for therapeutic applications.

## 1. Introduction

In the interdisciplinary fields of cellular biology and nanoengineering, a profound understanding of how cells migrate and interact with their microenvironments is pivotal [1,2]. This knowledge is not only critical for advancing regenerative medicine but also for developing targeted therapies for complex diseases such as cancer [3]. Directed cell migration, where cells move toward specific targets, plays important roles in numerous biological processes, including tissue repair, embryonic development, and immune responses [4,5,6]. The mechanisms of directed cell migration reveal a complex interplay between cells and their microenvironment, influenced by an array of physical and biochemical cues [7,8,9].

Recent advancements in nanoengineering have led to the development of sophisticated nanotopographies that mimic the natural microenvironment of the extracellular matrix (ECM) [10,11,12,13]. Cell growth and differentiation on nanoscale surfaces were mediated by changes in cell adhesion [14,15]. Nanostructures have been shown to influence cell morphology [16] and direct cell movements [17,18]. Rectangularly arranged nanopillars [19] and nanostructures in gratings [20] enhanced the directional formation of focal adhesions (FAs) and the organization of actin filaments, leading to biased cell movements. Asymmetric nanosawteeth induced spontaneous cell polarization and migration, leading to a unidirectional migration [21]. Additionally, nanotopographies regulate cell migration by directly affecting FAs protein and gene expression [22,23]. By replicating the physical complexity of the ECM, nanoengineered surfaces offer unprecedented opportunities to study and manipulate cell migration.

The metabolic process of cell migration, particularly the role of oxygen (O_2_) consumption, is crucial for the energy-enabled processes supporting cellular motility [24]. Detecting O_2_ in miniaturized platforms such as microfluidic devices, cell cultures, and tissues is important for understanding and controlling cell behaviors and viability [25,26]. Two primary sensing approaches, amperometric electrochemical sensing and luminescent optical sensing, are commonly employed for O_2_ detection in biological applications. Amperometric devices, often exemplified by Clark-type electrode sensors, are widely used to measure O_2_ concentration [27]. However, these sensors consume O_2_, which alters the levels exposed to cells. Moreover, integrating these probes into cell culture platforms for continuous measurement poses challenges [26,28,29]. Conversely, sensors based on optical excitation and detection of an embedded O_2_-sensitive dye offer a promising alternative [30]. These sensors function by measuring the quenching effect of O_2_ on the luminescence of the dyes, thus accurately inferring the O_2_ concentration [31]. Pt(II) and Pd(II) porphyrin complexes are particularly effective in live systems due to their high sensitivity to O_2_ concentration [28,32]. Utilizing platinum octaethylporphyrin ketone (PtOEPK) dye as an optical O_2_ sensor to measure temporal and spatial variations in O_2_ of single cells is advantageous. As cells traverse different engineered surfaces, they need to adapt their metabolism to meet the demands of movement. This adaption is especially pronounced when cells alter their migration direction, reflecting the intricate link between metabolic processes and directional changes. The PtOEPK dye sensor can quantify the O_2_ consumption when the cells change migration directions, which reflects the mitochondria activities and metabolic processes. Studying these dynamics are crucial for developing biomaterials that can precisely control cell movements for therapeutic purposes [5,33,34].

Previous research has demonstrated that cells align better along grooves compared to flat surfaces [4,35]. Cell guidance can be regulated by different designs [20,36] and dimensions [6,19] of nanostructures. Additionally, plasmonic sensors with nanopillars can be used as detectors for cell filopodia [37]. However, a comprehensive understanding of how nanotopographies specifically affect cell migration and physiological processes remains elusive. Furthermore, existing methodologies for real-time metabolic monitoring during cell migration are limited, posing a substantial barrier to in-depth understanding of the metabolic dynamics that influence cell migration direction [38]. This research aims to bridge these gaps by leveraging PtOEPK dye to monitor O_2_ consumption and correlate these fluorescence signals from the PtOEPK dye with changes in cell migration directions. By culturing MC3T3-E1 cells on platforms with nanotopography and applying live-cell imaging techniques, this study methodically analyzes how these nanotopographies influence cell motility, morphology, and metabolic responses over 16 h. The integration of PtOEPK dye with nanotopographies introduces a simple and novel method for the real-time monitoring of cellular metabolic responses and detecting cell migration directional changes. This O_2_ detection platform allows for the direct observation of O_2_ consumption when cells change migration directions. Understanding this metabolic process is crucial for developing treatments that target cellular metabolism, which is important in disease therapies. This approach can be extended to investigate other cell types and conditions, broadening its impact on personalized medicine and therapeutic innovations.

## 2. Materials and Methods 

### 2.1. Nanostructures Patterning on Grating Ridges by Nanoimprint Lithography

**Figure 1:**
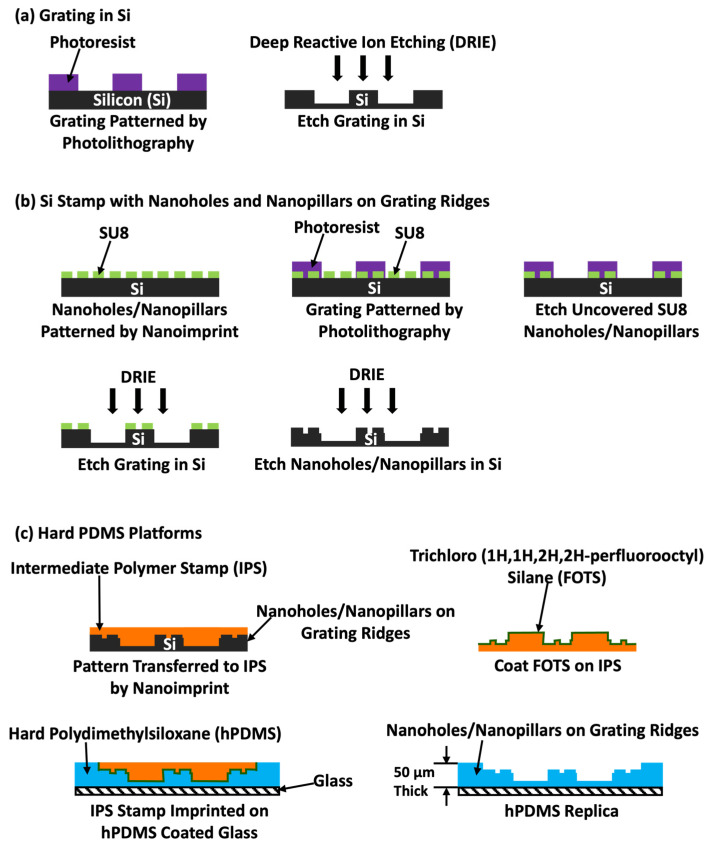
Fabrication technology of (**a**) grating, (**b**) nanoholes and nanopillars on grating ridges, and (**c**) hard polydimethylsiloxane replica.

Gratings were fabricated on a silicon (Si) substrate using photolithography and deep reactive ion etching (DRIE, LPX ICP-SR, SPTS, Newport, UK), as depicted in Figure 1a. SPR6112B positive photoresist was spin-coated on a Si substrate and exposed to ultraviolet (UV) light to generate gratings. The patterned Si substrate was then etched by DRIE, creating a Si stamp with 6/4 µm ridge width/trench width (R/T) and 4.5 µm deep gratings. To fabricate nanoholes and nanopillars on the grating ridges, a flexible intermediate polymer stamp (IPS) with 290 nm wide and 500 nm deep nanoholes and nanopillars was used for nanoimprint lithography (NIL, EITRE^®^ 6, Obducat, Lund, Sweden) [37,39]. The IPS was treated with trichloro(1H,1H,2H,2H-perfluorooctyl) silane (FOTS, J&K Scientific, San Jose, CA, USA) at 80 °C for 20 min to promote stamp separation after nanoimprinting. SU8 (Kayaku Advanced Materials, Westborough, MA, USA) nanoholes and nanopillars were first nanoimprinted on a Si substrate using the IPS stamp at 90 °C and 30 bar with UV exposure for 60 s, followed by hard bake at 150 °C for 10 min after demolding, as shown in Figure 1b. Next, a 1.7 μm thick SPR6112B positive photoresist was spin-coated on the SU8 nanoholes and nanopillars at 2500 rotations per minute (rpm) for 60 s. After UV exposure, the patterned gratings were formed on top of the nanoholes and nanopillars. An O_2_/SF_6_ plasma was used to remove the uncovered SU8 nanoholes and nanopillars by reactive ion etching under a condition of 20/2 sccm O_2_/SF_6_, 10 mTorr pressure, and 120 W RF power for 4 min. The patterned SPR6112R positive photoresist was used as an etch mask to etch 6/4 μm R/T and 4.5 μm deep gratings onto the Si. Subsequently, the nanoholes and nanopillars on the grating ridges as Si stamps were formed by etching in the DRIE system. 

To observe the cell migration on the designed patterns, the IPS nanoimprinting method was used to transfer the patterns from the IPS replicas to hard polydimethylsiloxane (hPDMS). In Figure 1c, the Si stamps were coated with FOTS and baked at 80 °C for 20 min to form an anti-stiction layer, followed by thermal NIL at 150 °C and 40 bar for 8 min to replicate the Si stamps into IPSs. These replicated IPSs were used to produce platforms with gratings, nanoholes on grating ridges, and nanopillars on grating ridges in hPDMS. hPDMS base and cross-linker (GELEST^®^, Morrisville, NC, USA) were mixed at a ratio of 1:1 and spin-coated on a cleaned glass substrate at a speed of 1000 rpm for 60 s, forming a 50 μm thick hPDMS layer. This hPDMS layer provided the mechanical stability and uniformity with the designed patterns for the study. Finally, the FOTS-coated IPS was faced down and imprinted on the hPDMS-coated glass and baked at 80 °C for 1 h. The hPDMS replicates were formed by peeling off the IPSs from the hPDMS layers. This method provided uniform and reproducible replicas in hPDMS platforms, ensuring consistent results with precisely controlled dimensions.

### 2.2. Cell Preparation and Time-Lapse Imaging

MC3T3-E1 mouse osteoblast cells were cultured in Dulbecco’s modified eagle medium with high glucose content (DMEM, Invitrogen, Carlsbad, CA, USA), under conditions of 37 °C and 5% CO_2_. The DMEM medium was supplemented with 10% fetal bovine serum (FBS, Gibco, Grand Island, NE, USA), 1% antibiotic-antimycotic (penicillin–streptomycin-glutamine, Gibco, Grand Island, NE, USA), and 1% alanyl-L-glutamine (GlutaMAX, Gibco, Grand Island, NE, USA). To ensure optimal cell health and growth, the culture medium was refreshed every 2 days, with cells being passaged upon reaching 80% confluency.

The glass slide with hPDMS platforms was bonded onto the bottom of O_2_ plasma treated, 35 mm diameter confocal dishes, followed by baking at 80 °C for 10 min. The hPDMS platforms were treated with an O_2_ plasma using 100 sccm O_2_ at 0.15 mbar pressure and 75 W RF power for 30 s to form a hydrophilic surface that promoted cell attachment to the surfaces. MC3T3-E1 cells were then added onto the hPDMS platforms at a density of 2.5 × 10^4^ cells/mL and incubated at 37 °C under 5% CO_2_ in a humidified incubator for 5 h. Time-lapse imaging was performed using an upright microscope (Eclipse Ni-E, Nikon, Tokyo, Japan) equipped with a 37 °C humidified incubator. To keep the cells healthy during the imaging, a CO_2_-independent medium (Invitrogen, Carlsbad, CA, USA) with 10% FBS, 1% GlutaMAX, and 1% penicillin-streptomycin-glutamine was used before the imaging. The images were captured at 5 min intervals over a 16 h period by a 20× magnification objective. All individual cells were tracked, and the cell migration speed and total migrating distance were analyzed by the manual tracking plugin in the ImageJ software (version 1.53v, National Institutes of Health, Bethesda, MD, USA).

### 2.3. Fixing Cells on Platforms with Gratings and Nanotopography 

MC3T3-E1 cells were fixed with 4% paraformaldehyde (Sigma-Aldrich, St. Louis, MO, USA) for 20 min at 22 °C after 22 h of incubation on the platforms. After the cells were fixed, the platforms were washed with 1% phosphate-buffered saline (PBS, Life Technologies, Carlsbad, CA, USA) twice. Subsequent dehydration of the cells was accomplished by immersing them in deionized (DI) water twice for 10 min each, followed by a series of ethanol solutions with concentrations of 30%, 50%, 70%, 80%, 90%, 95%, and 100%, with each solution applied for 5 min. The samples were supercritically dried using a critical point dryer (EM CPD300, Leica, Wetzlar, Germany) for 4 h. Before imaging, the dried samples were coated with a thin layer of gold to avoid charging. Imaging was carried out using a scanning electron microscope (SEM, SU5000, Hitachi, Chiyoda City, Japan). 

To quantify the cell morphology, the freehand selection tool of the ImageJ software was used to outline the cell, followed by the selection of parameters such as area and cell shape description.

### 2.4. F-Actin Structures in MC3T3-E1 Cells 

To stain the cells for fluorescent imaging, the fixed cells were first premetallized in 0.1% Triton X-100 (ThermoFisher, WA, USA) solution for 15 min before being immersed in a blocking solution of 1% bovine serum albumin (ThermoFisher, WA, USA) for 30 min. Cells were then incubated with Alexa Fluor 555 phalloidin (Sigma-Aldrich, St. Louis, MO, USA) for 2 h to stain F-actin. Following this, cells were washed with 1X PBS twice before being incubated with 4′,6′-diamidino-2-phenylindole (DAPI, Sigma-Aldrich, St. Louis, MO, USA) for 30 min to visualize the cell nuclei. The cells were subsequently washed and kept in 1X PBS for imaging. Confocal images were captured by a confocal inverted microscope (Stellaris 8, Leica, Wetzlar, Germany). Fluorescence signals of F-actin and nuclei were imaged by 512 and 405 nm laser, and marked by red and blue color, respectively. 

### 2.5. Platforms with PtOEPK Dye and hPDMS for O_2_ Sensing

To prepare the optical O_2_ sensor film, polystyrene (PS) pellets (Sigma-Aldrich, St. Louis, MO, USA) were dissolved in toluene (Sigma-Aldrich, St. Louis, MO, USA), creating a 2% weight/weight solution. This solution was subjected to an ultrasonic bath for 30 min to ensure complete dissolution. The resultant PS solution was then sealed in a glass bottle and stored at -20 °C to prevent toluene evaporation and maintain stability. Subsequently, PtOEPK dye (Frontier, Logan, UT, USA) was introduced to the PS solution at 1 mg/mL concentration. The PtOEPK/PS sensor film was generated by pipetting the solution onto the glass substrate and spin-coating at 900 rpm for 40 s, resulting in a uniform 600 nm thick layer. The PtOEPK dye and hPDMS layers allowed O_2_ diffusion through the hPDMS layer to reach the PtOEPK sensing layer for efficient O_2_ quenching of the luminescence. The 600 nm thick PtOEPK layer and the 50 μm thick hPDMS layer used in this study represent an optimized balance. This combination provides sufficient sensitivity for detecting O_2_ consumption by single cells while ensuring the mechanical stability and uniformity of the hPDMS layer. Although a thicker layer might enhance mechanical stability, it could reduce the sensitivity of O_2_ detection due to a longer O_2_ diffusion path and increased light scattering. 

**Figure 2:**
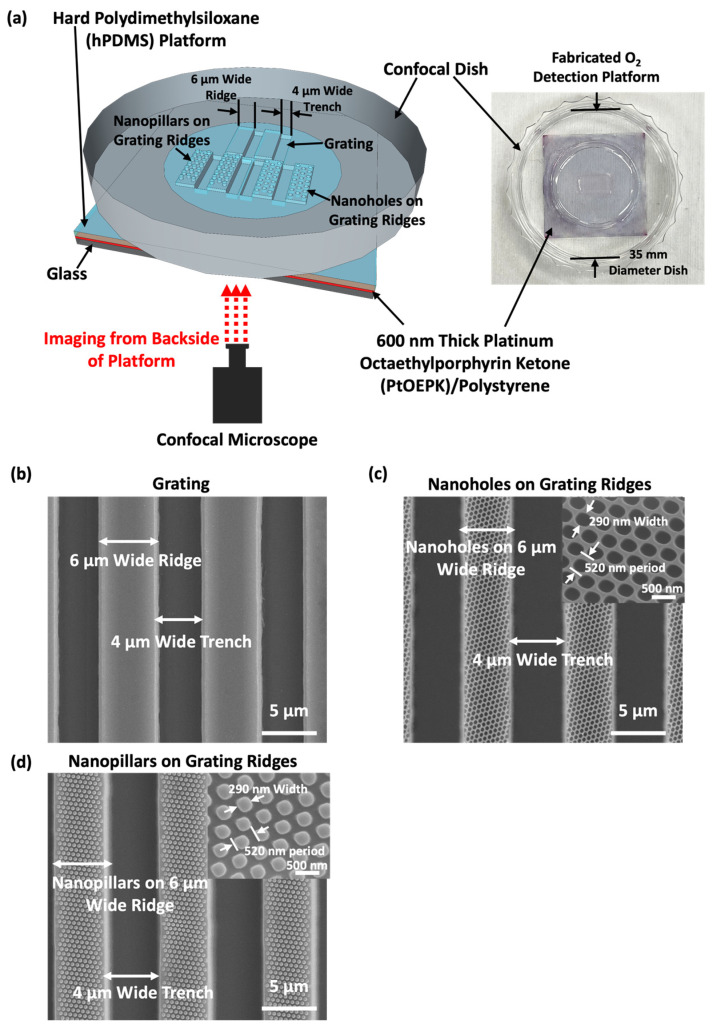
(**a**) Schematic of O_2_ detection setup. Scanning electron micrographs (SEMs) of (**b**) grating with 6/4 μm ridge width/trench width and 4.5 μm deep, (**c**) 280 nm wide and 500 nm deep nanoholes on ridges of grating, and (**d**) 280 nm wide and 500 nm deep nanopillars on ridges of grating.

Supplementary Figure S1 outlines the fabrication technology for the O_2_ detection platform. Firstly, the glass slide was cleaned with acetone, propanol, and DI water for 10 min each and then dried on the hot plate at 150 °C for 15 min, as shown in Supplementary Figure S1a. Subsequently, 75 µL PtOEPK/PS solution was spin-coated onto the glass slide, forming a 600 nm thick, smooth sensor layer, as shown in Supplementary Figure S1b. The sensor layer was then baked at 105 °C for 5 min and air dried. Platforms with gratings, nanoholes on grating ridges, and nanopillars on grating ridges were transferred by imprinting the IPS on hPDMS, as shown in Supplementary Figure S1c. After peeling off the IPS stamp from the hPDMS layer, the glass slide with a PtOEPK sensor layer and patterns in hPDMS was adhered to a confocal dish using an O_2_ plasma with 100 sccm O_2_ at 0.15 mbar pressure and 75 W RF power for 30 s, as shown in Supplementary Figure S1d. Figure 2a shows that the O_2_ detection platform consisted of a glass substrate, an optical sensor layer, and a hPDMS platform with gratings and/or nanotopography. Platforms were stored in the dark to prevent photobleaching and degradation of the optical O_2_ sensor, thus ensuring the accuracy and consistency of O_2_ detection.

The PtOEPK dye was chosen to observe O_2_ consumption around single cells with different migration behaviors, due to its high sensitivity to O_2_ concentration changes and ability to be reversely quenched [25,27,29,40,41]. PtOEPK dye exhibits superior stability compared to other O_2_-sensitive dyes and it can withstand prolonged exposure to light without significant degradation or loss of fluorescence intensity [29,42]. This robustness is particularly crucial for the extended 16 h time-lapse imaging experiments used in this study. When imaging O_2_ consumption around single cells, PtOEPK dye interacted with the laser source due to the porphyrin ring in its molecular framework [30]. This ring absorbs light at 615 nm, energizing the molecule and subsequently emitting red light within the 650–800 nm range during relaxation. The O_2_ consumption was related to the PtOEPK dye intensity, showing high PtOEPK dye intensity corresponds to low O_2_ level on the film, as well as high O_2_ consumption by cells. This emission was detected by a confocal microscope, serving as a reliable O_2_ concentration mapping that is related to the O_2_ consumption of cells. 

To reveal the relationship between O_2_ consumption and cell movements, three distinct guiding patterns with/without nanotopography were designed to direct cell movements, including gratings, nanoholes on grating ridges, and nanopillars on grating ridges, as shown in Figure 2b. All gratings were 6/4 µm R/T and 4.5 µm deep. The nanoholes and nanopillars on the grating ridges were 290 nm wide and 500 nm deep with a 520 nm period. 

### 2.6. Live Cell Tracking of Mitochondria and PtOEPK Dye Signals 

The cells were stained with MitoTracker green (Invitrogen, Carlsbad, CA, USA), a specific mitochondrial dye, to visualize the presence of mitochondrion in the cells. The dye was diluted in DMEM at a ratio of 1:200, followed by an incubation period of 15 min at 37 °C. Cells were then washed thrice with 1× PBS, and DMEM was added to the dish for time-lapse imaging. 

A confocal microscope with an inverted 20× objective lens was used for image acquisition, and the imaging was performed from the backside of the platforms. The laser source with excitation wavelengths of the PtOEPK dye at 615 nm and the MitoTracker dye at 488 nm incident on the O_2_ detection platforms from below. Upon reaching the detection platforms, the light is absorbed by the fluorescent molecules of the PtOEPK dye and MitoTracker green dye and re-emitted at longer emission wavelengths from 650 to 800 nm for the PtOEPK dye and from 490 to 550 nm for the MitoTracker green dye. The re-emitted light is collected through a 20× objective lens and analyzed for their changes during cell migration.

## 3. Results and Discussion

### 3.1. Protrusion and Filopodium Formation on Platforms with Nanotopography 

**Figure 3:**
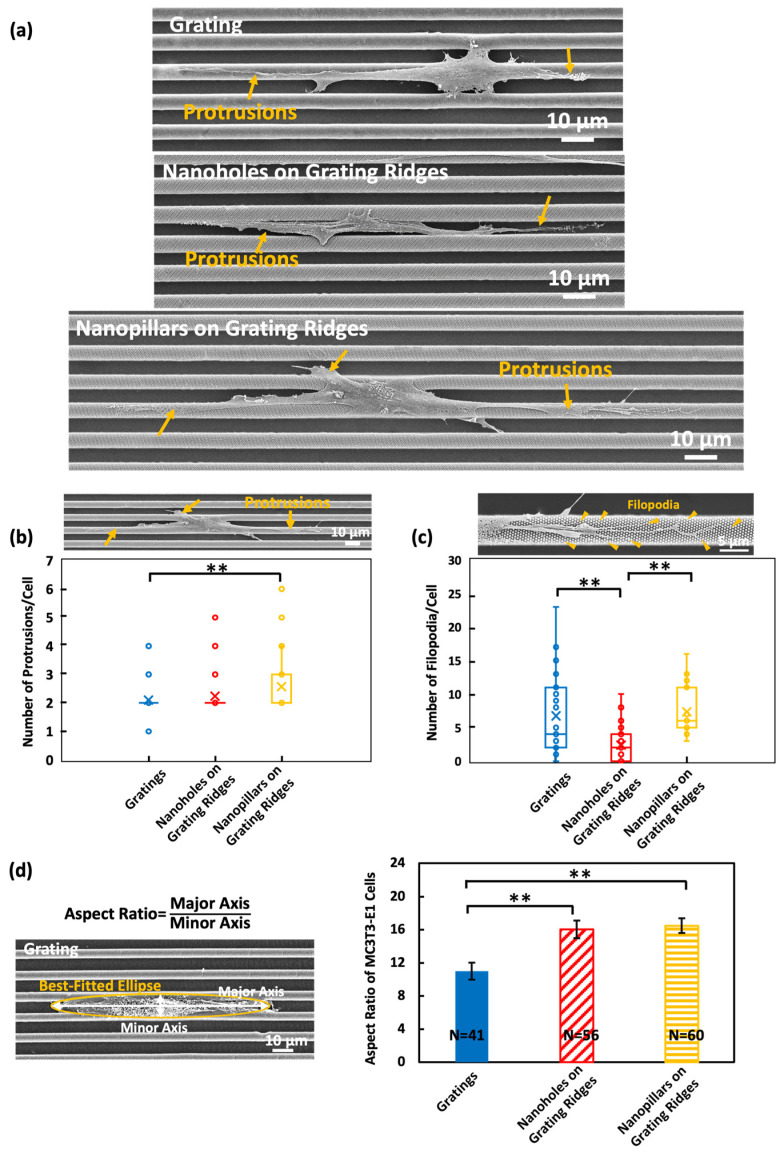
(**a**) SEMs of MC3T3-E1 cells migrated on surfaces of grating, nanoholes on grating ridges, and nanopillars on grating ridges. (**b**) Numbers of protrusions per cell, (**c**) filopodia per cell, and (**d**) cell aspect ratio analysis from SEMs. Mean values are represented by × and median values are represented by–in (**b**,**c**). One-way ANOVA with Tukey’s post hoc test, ** *p* < 0.01.

It has been reported that nanotopography can lead to cell guidance and influence cell morphology [20,21,36]. Images were captured to investigate how fixed MC3T3-E1 cells adopted their shapes in response to platforms with different topographies. In Figure 3a, MC3T3-E1 cells were found to align with the grating orientation and displayed an elongated cell shape on the grating [8]. Cells were mostly found in the grating trenches when nanoholes were patterned on the grating ridges, with small parts of cells attached to the nanoholes. On the surface with nanopillars on the grating ridges, cells showed more protrusions and filopodia extending on the nanopillar surface.

Based on the micrographs, the number of protrusions and filopodia of MC3T3-E3 cells on various surfaces were analyzed. Protrusions are extensions of the cell membrane, with a width larger than 400 nm and a length of 5–50 μm, and are related to how cells explore the surrounding microenvironments. Filopodia are thin extensions from the protrusions with a width of 200–400 nm and are rich in actin filaments, which promote FA formation in cells and enhance cell movements. It was found that cells cultured on the nanopillars over grating ridges exhibited an increased number of protrusions and filopodia, with an average of 2.6 protrusions/cell and 7.3 filopodia/cell, as shown in Figure 3b,c. The increased protrusions and filopodia suggest cells have an enhanced contact area on the surface with nanopillars, improving the attachments between the cells and the nanopillars. This increased contact area amplified the ability of cells to sense the top and sidewall surfaces of the nanopillars. In contrast, cells on the nanoholes showed fewer protrusions and filopodia, with 2.2 protrusions/cell and 2.4 filopodia/cell. Cells were mostly found in the grating trenches with nanoholes on top of the grating ridges. Compared to the nanopillars, it was more difficult for protrusions and filopodia to form inside the nanoholes, resulting in fewer protrusions and filopodia attached to the nanoholes over grating ridges. Consistent with previous findings, 2.1 protrusions/cell and 6.7 filopodia/cell were found when cells were on gratings [20]. 

The aspect ratio of the cells, defined as the ratio of the major axis to the minor axis of the best-fitted ellipse to each cell, varied significantly across the different platforms, as shown in Figure 3d. Cells cultured on the nanoholes and nanopillars over grating ridges exhibited a higher aspect ratio of 16.0 and 16.5, respectively, indicative of a more elongated cell shape. Nanopillars on the grating ridges promoted the extension of long protrusions and filopodia, aiding in cell exploration and elongation. As cells were mostly found in the grating trenches with nanoholes on the grating ridges, cells contacted the sidewalls of the grating trenches, which facilitated cell stretching and elongation. A lower aspect ratio of 10.1 was observed when cells migrated on the gratings, as some cells bridged on the top and across several grating ridges, which resulted in larger minor axes and lower aspect ratios.

These findings show the profound impact of nanotopographies on the morphological characteristics of MC3T3-E1 cells. The increased number of protrusions and filopodia in cells on the surface of nanopillars on grating ridges not only reflects an enhanced ability of cells to contact the top and sidewalls of the nanopillars but also suggests potential improvement in cell migration and adhesion. The observed elongated cell shapes on the nanohole and nanopillar surfaces allowed cells to establish the cell polarity along the intended direction of migration. Moreover, the elongated cells tend to form elongated FAs, which provide traction for cell migration and orient cells along the elongated axis compared to the rounded cells. Therefore, the nanotopographies, especially nanopillars on grating ridges, offer promising avenues for enhancing cell motility and guiding cell movements [19].

### 3.2. Dotted F-Actin Structures in MC3T3-E1 Cells on Nanohole and Nanopillar Surfaces

**Figure 4:**
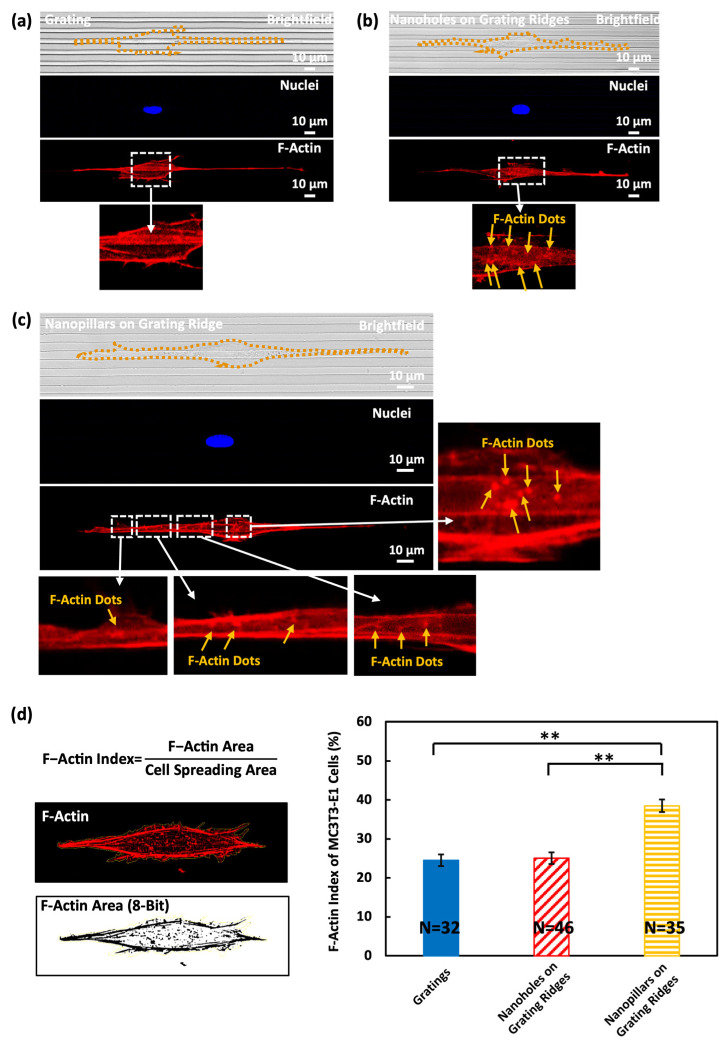
Brightfield and fluorescent imaging of MC3T3-E1 cells on surfaces of (**a**) grating, (**b**) nanoholes on grating ridges, and (**c**) nanopillars on grating ridges. (**d**) F-actin index of MC3T3-E1 cells on surfaces of gratings, nanoholes on grating ridges, and nanopillars on grating ridges. One-way ANOVA with Tukey’s post hoc test, ** *p* < 0.01.

The protrusions and filopodia are rich in actin filaments, which facilitate the formation of adhesion sites. Figure 4a–c detail the distribution of F-actin within MC3T3-E1 cells, which is critical for maintaining cell structure and improving cell movements. The cells were stained with phalloidin and DAPI, exhibiting F-actin in red and the nucleus in blue. In Figure 4a, brightfield and fluorescence images revealed that cells on grating exhibit a stretched and elongated morphology, with actin stress fibers aligned along the grating orientation. These aligned actin stress fibers generated the necessary force for cells to move along the gratings [8]. When nanostructures were patterned on the grating ridges, in addition to the aligned actin stress fibers, dotted F-actin structures were found around nanoholes and nanopillars, as shown in Figure 4b,c. When cells were on the nanoholes over grating ridges, the dotted F-actin structures were formed inside the nanoholes and partially trapped by the nanoholes. The nanoholes acted as trapping sites that confined the protrusions and filopodia of cells, making them more difficult to move forward. Also, the nanoholes disrupted the formation of continuous adhesion sites, which hindered cell movements. Notably, more dotted F-actin structures were observed on the nanopillars, as illustrated in Figure 4c, suggesting cells maximized their surface contact areas and formed additional adhesion sites on the top and sidewalls of the nanopillars [19]. These additional adhesion sites around the nanopillars promoted the formation of protrusions and filopodia without trapping them, enhancing cell movements.

These findings were verified by observing the F-actin distribution of MC3T3-E1 cells on nanopillars in the vertical direction. Supplementary Figure S2 reveals the vertical alignment of F-actin along the sidewalls of nanopillars. By stratifying the fluorescence images of the cell into several depths from the top surface to 2.4 µm below, the fluorescence intensity of F-actin changed with the depth of nanopillars, as shown in Supplementary Figure S2a. At the nanopillar surface, the F-actin fluorescence intensity was lower, and it gradually increased with the depth of nanopillars down to 1.2 µm. This suggests a dense aggregation of F-actin along the sidewalls of nanopillars. The fluorescence intensity declined at larger depths, with the lowest levels observed at 2.4 µm below the top surface, which was the bottom of the nanopillars. These observations reflect a depth-dependent F-actin distribution on surfaces with nanostructures, which agrees with the findings that F-actin of cells was observed in the vertical direction of nanopillars [19]. In Supplementary Figure S2b, three regions of the nanopillar surface marked by A, B, and C were analyzed to show the depth-dependent distribution of F-actin on nanopillars. Each region demonstrated a similar trend in F-actin fluorescence intensity variation with depth, although the peak values varied, reflecting cells attached to the sidewalls of the nanopillars at different depths. Such findings emphasize the potential of nanopillar-engineered surfaces to influence not only cell morphology but also intracellular organization, which is pivotal for understanding cell mechanics and developing biomaterials for tissue engineering and regenerative medicine. 

The quantification of F-actin areas expressed as the F-actin index was measured as the ratio of F-actin area to cell spreading area. The cells on the nanopillars over grating ridges exhibited the highest percentage of F-actin area of 38.5%, indicating a higher density of actin filaments per cell area, which is consistent with the observation from Figure 4c. However, on the gratings and nanoholes over grating ridges, the F-actin index was 24.5% and 25.0%, respectively. The varied F-actin areas affect cell migration behaviors. F-actin is the key component of the cytoskeleton and provides mechanical support and facilitates shape changes during the movements. The increased F-actin area indicates a higher level of actin polymerization, which enables faster assembly and disassembly of actin filaments during cell movements. For an effective cell migration directional change, F-actin will re-establish polarity so that the cells can reorient themselves. The denser F-actin structure observed on nanopillars allows faster cytoskeletal reorganization and remodeling of its shapes when cells change their migration directions [43,44].

### 3.3. Nanopillars Enhanced Cell Motility and Induced Migration Directional Changes

**Figure 5:**
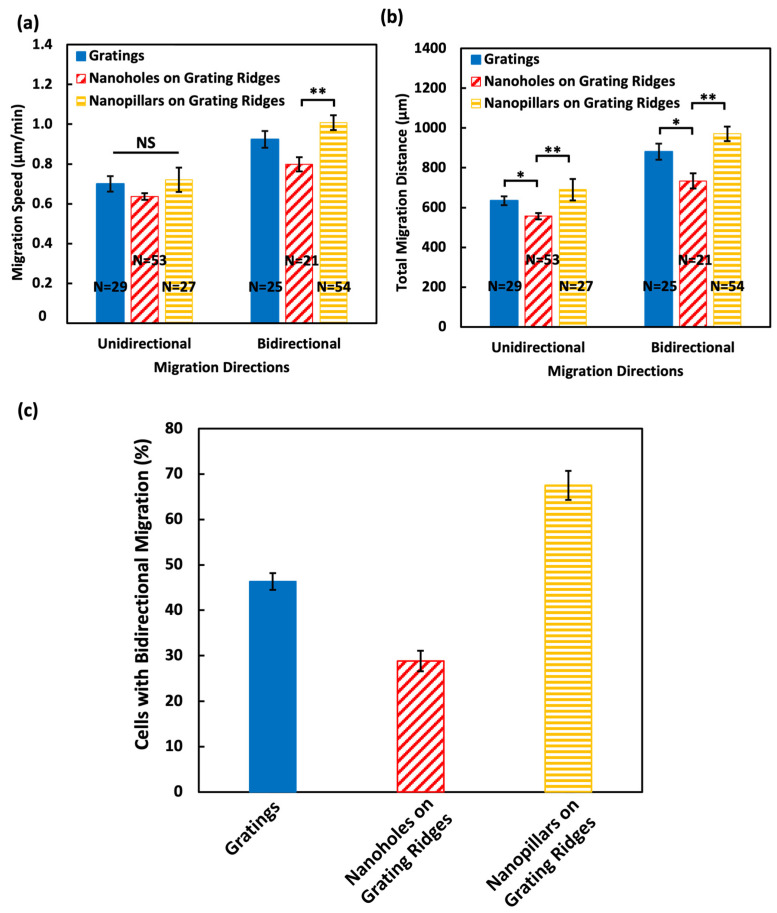
(**a**) Migration speed, (**b**) total migration distance, and (**c**) percentage of MC3T3-E1 cells that changed migrating directions on surfaces of gratings, nanoholes on grating ridges, and nanopillars on grating ridges during 16 h. One-way ANOVA with Tukey’s post hoc test, NS—not significant, * *p* < 0.05, and ** *p* < 0.01.

F-actin within the protrusions and filopodia enhances the formation of adhesion sites and promotes cell movements. Using time-lapse optical microscopy, cell motility was assessed by measuring the migration speed and total migrating distance covered by MC3T3-E1 cells over time. The cells were seeded evenly on the platforms. Figure 5a presents the average migration speed of MC3T3-E1 cells across different platforms. Consistent with the previous studies, MC3T3-E1 cells were guided by gratings [7,8,9], moving at a speed of 0.7 μm/min in unidirectional and 0.92 μm/min in bidirectional cell migration. Notably, the cells on nanopillars over grating ridges exhibited the highest speed of 1.0 μm/min in bidirectional migration, suggesting that the nanopillars enhanced cell movements. Conversely, cells migrated at a lower speed of 0.6 μm/min in the unidirectional and 0.8 μm/min in the bidirectional cell migration when nanoholes were formed on top of the grating ridges, indicating that the partial confinement of protrusions and filopodia inside the nanoholes was an impediment to cell migration [20]. 

The total migration distance of the cells over 16 h, as shown in Figure 5b, further elucidates the impact of topographical variations. Cells migrated over longer distances when they were in the bidirectional migrations compared to the unidirectional migrations. When cells moved unidirectionally on the surfaces of gratings, nanoholes on grating ridges, and nanopillars on grating ridges, the total migrating distance was 634, 556, and 690 μm, respectively. A longer distance of 881, 733, and 970 μm was measured when cells migrated bidirectionally across surfaces of gratings, nanoholes on grating ridges, and nanopillars on grating ridges, respectively. Taken together, the cells on nanopillars not only moved faster but also covered significantly longer distances. The sidewalls of the nanopillars provided more surface contact areas for cells to attach. Additionally, the increased protrusions and filopodia allowed cells to form more adhesion sites not only on the top but also on the sidewalls of nanopillars [20,45]. These contributed to the observed faster migration speed and longer migrating distances. In contrast, the migration speed and total distance covered by the cells on nanoholes were comparatively slower and shorter, indicating that the partial trapping of cell protrusions and filopodia by nanoholes may impede the ability of movements [20].

Figure 5c quantifies the percentage of cells that moved bidirectionally during 16 h. A total of 46.3% of MC3T3-E1 cells moved bidirectionally, with changes in migration direction, over 16 h. The nanopillars on grating ridges induced cells to change their migration directions more frequently, with a probability of 67.5%. Typically, gratings can guide cells to migrate along the grating orientation. However, the nanopillars introduced additional surface topography for cell interactions. This led to an increased formation of protrusions and filopodia to sense and form more adhesion sites on nanopillars with more dotted F-actin structures along the sidewalls of the nanopillars. These denser F-actin structures provided more active cytoskeletal reorganization within the cells. The dynamic changes in the cytoskeleton could support frequent changes in the orientation of polarized cells, thereby causing directional changes [43,44]. Moreover, the tendency of the cells to align in the directions of the two orthogonal axes of the nanopillar arrangement provided additional direction cues for the cells to migrate [19]. Conversely, only 28.8% of cells on the surface of nanoholes on grating ridges changed their migration direction, as the partial trapping of protrusions and filopodia in nanoholes hindered cell movements and caused a limited migrating distance. 

### 3.4. Correlation between Mitochondria and PtOEPK Dye Signals

In this study, it has been demonstrated that nanotopography could effectively change cell migration directions. Next, the relationship between O_2_ consumption and cell migration directional changes was investigated. The PtOEPK dye was chosen for its high sensitivity to O_2_ concentration changes and its ability to be reversely quenched, which makes it ideal for real-time monitoring of cellular O_2_ consumption. Unlike previous studies that utilized PtOEPK dye primarily as an indicator of O_2_ levels in the cell environment, this research demonstrates its application in observing single-cell behaviors and correlating them with mitochondrial activities. This novel use of PtOEPK dye allowed for the detection of dynamic metabolic responses related to O_2_ consumption at the single-cell level, providing insights into the intricate relationship between cell migration and metabolism. This method bridges the gap between cell migration studies and cellular metabolism, offering high sensitivity and specificity in detecting minute changes in O_2_ concentration.

**Figure 6:**
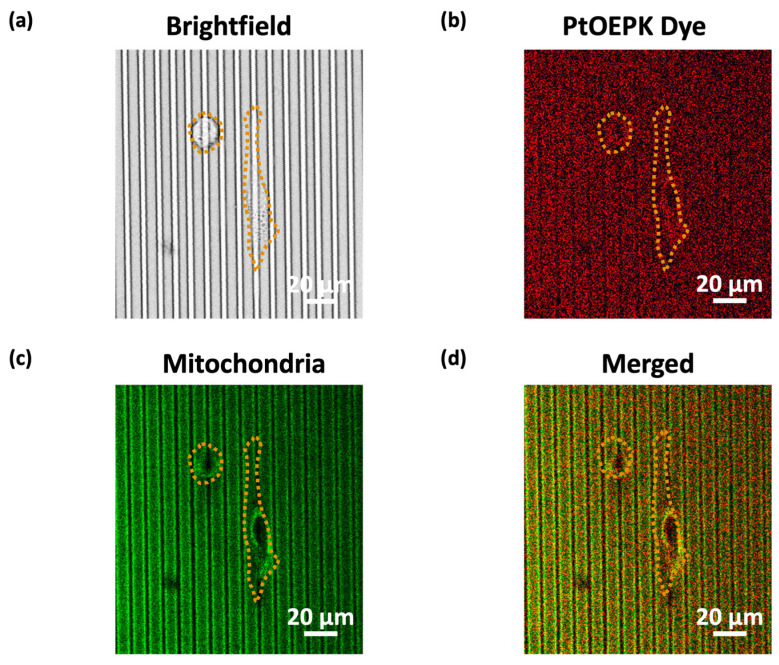
(**a**) Brightfield image. Fluorescence signals of (**b**) platinum octaethylporphyrin ketone (PtOEPK) dye, (**c**) mitochondria, and (**d**) merged images of cells.

Figure 6a–d display the brightfield and fluorescence images of MC3T3-E1 cells on a grating. The yellow dotted line outlined the cell shape. The O_2_ consumption was characterized by the intensity of PtOEPK O_2_-sensitive dye, which emitted red light after the porphyrin ring absorbed the light energy [30], as shown in Figure 6b. To monitor the mitochondria of MC3T3-E1 cells, cells were incubated with MitoTracker Green before imaging, resulting in green light emission in the range of 490–550 nm, as shown in Figure 6c. The resulting images showed colocalization between the position of the mitochondria and the PtOEPK dye signal, independent of the cell shape. This suggests a correlation between mitochondria activities and PtOEPK dye intensity, which is an indication of O_2_ consumption, as illustrated in Figure 6d. 

Mitochondria, intracellular organelles distributed throughout the cytoplasm surrounding the nucleus, play a pivotal role in energy provision for cell migration [46,47]. This process involves O_2_ consumption, which is a key element to energy production within mitochondria. Mitochondria principally meet the energy requirement for cell migration through the production of adenosine triphosphate (ATP) via oxidative phosphorylation, a process that requires O_2_. The ATP thus generated powers actin polymerization, membrane extension, and cell movements during migration. As such, the availability of O_2_ is vital for efficient ATP production [34,47,48]. In addition to energy production, mitochondria contribute to cell migration via reactive O_2_ species (ROS) signaling. While excessive ROS levels can be deleterious, moderate levels play regulatory roles in migration. Mitochondria generate ROS as byproducts of oxidative phosphorylation and regulate ROS levels [49,50]. Cell migration also involves metabolic adaptation as cells reconfigure pathways to meet the energy demands of the migration [34]. Therefore, mitochondria, O_2_ consumption, and cell migration are closely interconnected. Mitochondria supply ATP for cell migration, and their O_2_ consumption influences ATP production and cell migration. Furthermore, mitochondria contribute to ROS signaling, metabolic reprogramming, and maintaining the overall energy balance required for the dynamic process of cell migration.

This study is the first to correlate mitochondria activities during cell movements with changes in PtOEPK dye intensity. A higher intensity of PtOEPK dye indicates a lower O_2_ concentration on the surface, corresponding to a higher O_2_ consumption. Thus, the high PtOEPK dye signal reflected regions of high mitochondria activities and O_2_ consumption during cell migration. Regardless of the cell shape, there was a consistent overlap between the PtOEPK dye and the mitochondria signal, as shown in Figure 6a–d. Supplementary Video S1 also supported this finding, showing simultaneous changes in the PtOEPK dye and mitochondria signals as MC3T3-E1 cells moved on the surface of nanopillars on grating ridges. Given that mitochondria are responsible for O_2_-driven energy production, regions with active mitochondria are directly related to higher O_2_ consumption, hence the higher PtOEPK dye luminescence. This localization correlation enhances our understanding of the spatial distribution of mitochondria and their role in the O_2_ consumption of MC3T3-E1 cells during migration, indicating a linkage between the measured PtOEPK dye intensity and mitochondria activities.

Various approaches have been developed to investigate cell migration, each with distinct advantages and limitations. Traditional two-dimensional (2D) cell cultures on flat substrates have been widely used due to their simplicity and ease of imaging. However, they fail to mimic the complexity of the three-dimensional (3D) ECM found in vivo [9,51]. Three-dimensional culture models, such as spheroids and organoids, provide a more physiologically relevant environment, allowing cells to interact in all dimensions. Despite their relevance, 3D models often suffer from limitations in imaging and quantification of cell behaviors due to the opacity and thickness of the constructs [51,52]. Microfluidic platforms also enable precise control over the cellular microenvironment, allowing for the study of cell migration under various chemical and physical gradients [53]. These devices can simulate the vascular environment and study the response of cells to shear stress and chemotactic gradients [54]. However, microfluidic systems can be complex to fabricate and operate, and they often require specialized equipment for analysis [55,56].

Nanotopographies, as demonstrated in this study, offer a unique approach to guide cell migration by providing physical cues that mimic the ECM microenvironment. Nanostructures such as nanopillars and nanoholes can influence cell adhesion, morphology, and migration direction by modulating focal adhesions and cytoskeletal organization. This study integrated O_2_ sensors with nanotopographies and enables real-time monitoring of cellular metabolic responses due to directional changes in migration. This method provides high spatial and temporal resolution, facilitating a deeper understanding of the relationship between cell migration and mitochondria activities. The O_2_ detection platform developed in this study addresses several limitations of existing methodologies. By combining nanotopographical cues with O_2_ biosensors, it offers a dual approach to studying cell migration. The nanostructures over grating ridges guide cell movements, while the O_2_ sensors provide real-time monitoring of cellular metabolism. This integrated approach enables the study of how changes in cell migration direction are coupled with metabolic demands, offering insights that are not accessible with traditional methods. Furthermore, the O_2_ detection platform is capable of distinguishing bidirectional cell migration and correlating it with metabolic activities, which presents a significant advancement in understanding the dynamics of cell movement.

### 3.5. O_2_ sensing of Single Cells with Unidirectional and Bidirectional Migrations

Nanotopography was patterned on grating ridges to modify cell migration, and the relationship between O_2_ consumption and cells with unidirectional and bidirectional migrations was analyzed. This method not only measures the O_2_ consumption but also provides insights into mitochondrial activities within individual cells, bridging the gap between cell migration studies and cellular metabolism. The PtOEPK dye exhibited high sensitivity and specificity, allowing the precise detection of minute changes in O_2_ concentration at the single-cell level. Bidirectional migrations present a stark contrast in the variation of PtOEPK dye intensity, with peaks formed in the PtOEPK dye intensity over time when cells changed migration directions. These peaks corresponded to the increased O_2_ consumption and the metabolic challenge posed by cells changing migration directions. The response time of the sensor is rapid, with changes in fluorescence intensity observable within minutes of O_2_ concentration changes. The PtOEPK dye was stable and robust under prolonged exposure to light with a consistent performance over 16 h. Cells on nanopillars exhibited a greater number of peaks in the PtOEPK dye intensity, as cells changed their migration directions more frequently on nanopillars over grating ridges, indicative of the increased metabolic response due to changes in migration directions [33]. The peaks in the PtOEPK dye intensity pointed to a dynamic metabolic adaptation process, as cells were likely to expend more energy to reorient and migrate when changing migration direction [5,34]. Bidirectional migration necessitates continuous adjustments in cell polarity and cytoskeletal organization, leading to increased O_2_ consumption [34,48,57]. Cells on nanopillars further accentuated this effect by experiencing more directional changes, resulting in more peaks in the PtOEPK dye intensity.

This study provides a robust and sensitive platform for the real-time, high-resolution monitoring of O_2_ consumption in single cells during migration. This approach enhances the understanding of the metabolic dynamics associated with cell migration, particularly the impact of directional changes on O_2_ consumption, offering new insights into cellular behaviors and metabolism.

**Figure 7:**
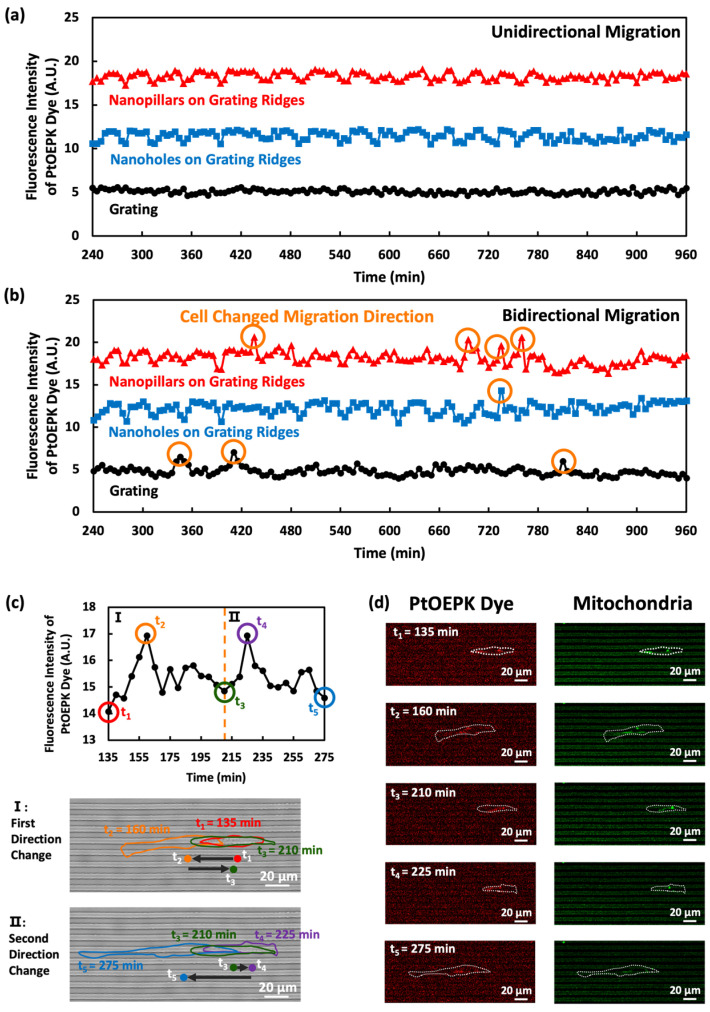
Changes in fluorescence intensity of PtOEPK dye around MC3T3-E1 cells during migration. (**a**) Unidirectional and (**b**) bidirectional cell migration on surfaces of gratings, nanoholes on grating ridges, and nanopillars on grating ridges. (**c**) Fluorescence intensity of PtOEPK dye over time during migration direction changes on surface of nanopillars on grating ridges. Colored dots indicate cell positions and arrows indicate direction of migration. (**d**) Fluorescence signals of PtOEPK dye and mitochondria of MC3T3-E1 cell varied as it changed migration directions.

Figure 7 shows the O_2_ consumption associated with cell migration across gratings, nanoholes on grating ridges, and nanopillars on grating ridges. In unidirectional migrations, where cells migrated only in one direction, the variations in PtOEPK dye fluorescence intensity were small across all surfaces, as shown in Figure 7a. This indicates the high accuracy and precision of the measurements. The total O_2_ consumption varies significantly between the different modes of migration. Specifically, bidirectional migration induced higher O_2_ consumption due to the increased metabolic demand associated with directional changes, as indicated by the peaks of the PtOEPK dye fluorescence intensity shown in Figure 7b.

Figure 7c shows the detailed changes in the PtOEPK dye intensity over time when a cell changed its migration direction twice. From t_1_ to t_2_, the cell migrated from right to left. At t_2_, the cell changed migration direction and a peak in PtOEPK dye intensity was detected. This fluorescence at t_2_ corresponds to the migration directional change, as well as the cytoskeleton reorganization, which is essential for changing the migration direction [33,34,58]. Further changes were associated with another intensity peak, from t_3_ to t_5_, as the cell moved from left to right. Each directional change induced a fluorescence intensity peak and could be used as a unique metabolic footprint. Based on the working principle of the PtOEPK dye, a higher PtOEPK dye intensity reflects a lower O_2_ concentration on the surface and, hence, increased O_2_ consumption. Each directional change required more energy for cytoskeletal reorganization and polarity adjustment, leading to greater O_2_ use. Figure 7d further contextualizes the PtOEPK dye intensity changes by correlating them with the mitochondrion dynamics. As the cell changed its migration direction twice from t_1_ to t_5_, there were notable changes in mitochondria distribution and activities within the cell. The changes in the PtOEPK dye signals followed the mitochondria signals, emphasizing the critical role of mitochondria in O_2_ consumption and energy production during cell migration directional changes [34].

## 4. Conclusions

In this study, O_2_ detection platforms were designed to realize the real-time monitoring of O_2_ consumption during unidirectional and bidirectional cell migrations. Gratings and gratings with nanotopographies were utilized to change cell migration directions. It was found that nanopillars on grating ridges significantly enhance cell motility and induce more frequent changes in cell migration directions. This enhancement is closely associated with the increased number of protrusions and filopodia, as well as denser F-actin structures. The increased number of protrusions and filopodia allowed cells to form additional adhesion points on the top and sidewalls of the nanopillars, which enhanced cell movements with a faster migration speed and longer migrating distance. The denser F-actin structures supported the faster cytoskeleton reorganization and remolding of cell shapes, aiding in directional changes during migration. Another key finding is the association between cell directional changes and O_2_ consumption, as detected by the PtOEPK dye sensor. Moreover, the PtOEPK dye signals followed mitochondria signals during cell migration, which is an indication of energy production and O_2_ consumption. By integrating the O_2_ sensor with nanotopographies, it was observed that PtOEPK dye fluorescence intensity formed peaks when cells changed migration directions. These peaks in PtOEPK dye intensity were observed when cells changed migration direction, indicating a higher O_2_ consumption. 

The results shed light on the dynamic interactions between MC3T3-E1 cells and their nanoengineered microenvironments, revealing how topographical cues can guide cell migration direction and influence O_2_ consumption. Integrating advanced nanoengineering platforms with an O_2_-consumption monitoring sensor provides new insights into designing biomaterials that can precisely modulate cell movements, offering potential breakthroughs in medical applications. This research represents a significant advancement in precision cell biology and tissue engineering. By decoding the intricate mechanisms of directed cell migration and its associated O_2_ consumption, this study contributes to the development of biomaterials that can foster specific cell behaviors to enhance therapeutic discovery. For example, in regenerative medicine, utilizing nanotopographical cues to enhance cell motility and direct migration can lead to the development of advanced scaffolds that promote faster and more organized tissue regeneration. Varying the arrangement and density of nanopillars on ridges creates gradients that guide cells in specific directions, potentially achieving more directional and predictable migration. 

Applying nanotopographies to various implant materials and designs could improve cell coverage and promote better integration with surrounding tissue. Specifically, investigating the optimal pattern dimensions and densities that maximize cell adhesion and proliferation on implant surfaces could lead to improved outcomes in implantable devices. In cancer research, exploring how different nanopattern densities and designs affect the migration of cancer cells compared to healthy cells could provide insights into metastasis and invasion mechanisms. This knowledge could lead to the development of surfaces that selectively inhibit cancer cell migration while promoting the migration of healthy cells, offering a novel approach to cancer treatment. Furthermore, the real-time monitoring of O_2_ consumption introduced by this study adds a novel dimension to our understanding of cell metabolism and migration dynamics. Developing biosensing platforms that simultaneously measure O_2_ consumption, glucose levels, pH changes, and other metabolic indicators could provide a comprehensive understanding of cellular responses to treatments. These platforms could be used in both research and clinical settings to monitor cellular responses to therapies, providing valuable information for personalized medicine. Ultimately, these findings have the potential to direct tissue engineering strategies and the design of targeted therapies, thereby leading toward more effective and personalized treatment modalities.

## Data Availability

Data are contained within the article and Supplementary Materials.

## Supplementary Materials

The following supporting information can be downloaded at: https://www.mdpi.com/article/10.3390/bios14080389/s1, Supplementary Figure S1: fabrication technology of pattern hPDMS guiding platform for O_2_ detection during cell migration; Supplementary Figure S2: Depth distribution of F-actin of MC3T3-E1 cell on nanopillars. (a) Fluorescence images of F-actin from 0 to 2.4 μm below the cell top surface. (b) Fluorescence intensity of three F-actin dots as a function of depth from cell top surface. Supplementary Video S1: time-lapse of MC3T3-E1 cell migrated bidirectionally on the surface of nanopillars on grating ridges.
