# Supplementary material for: Detecting Nanotopography Induced Changes in Cell Migration Directions Using Oxygen Sensors
Source: Biosensors (Basel). 2024 Aug 12;14(8):389. doi: 10.3390/bios14080389 (PMC11352363; doi:10.3390/bios14080389)
Supplement: Supplementary file 1 [file biosensors-14-00389-s001.zip › Nanotopography-Supplementary Materials-7.2.24.pdf]

**Supplementary Information for:**

# Detecting Nanotopography Induced Changes in Cell Migration Directions Using Oxygen Sensors

Muting Wang and Stella W. Pang *
Department of Electrical Engineering
Centre for Biosystems, Neuroscience, and Nanotechnology
City University of Hong Kong, Hong Kong, China

*Corresponding Author:
Stella W. Pang (pang@cityu.edu.hk)
Department of Electrical Engineering
City University of Hong Kong
83 Tat Chee Avenue, Kowloon
Hong Kong, China
Phone: +852 3442 9853
Fax: +852 3442 0562

## Supplementary Figures and Supplementary Figure Legends

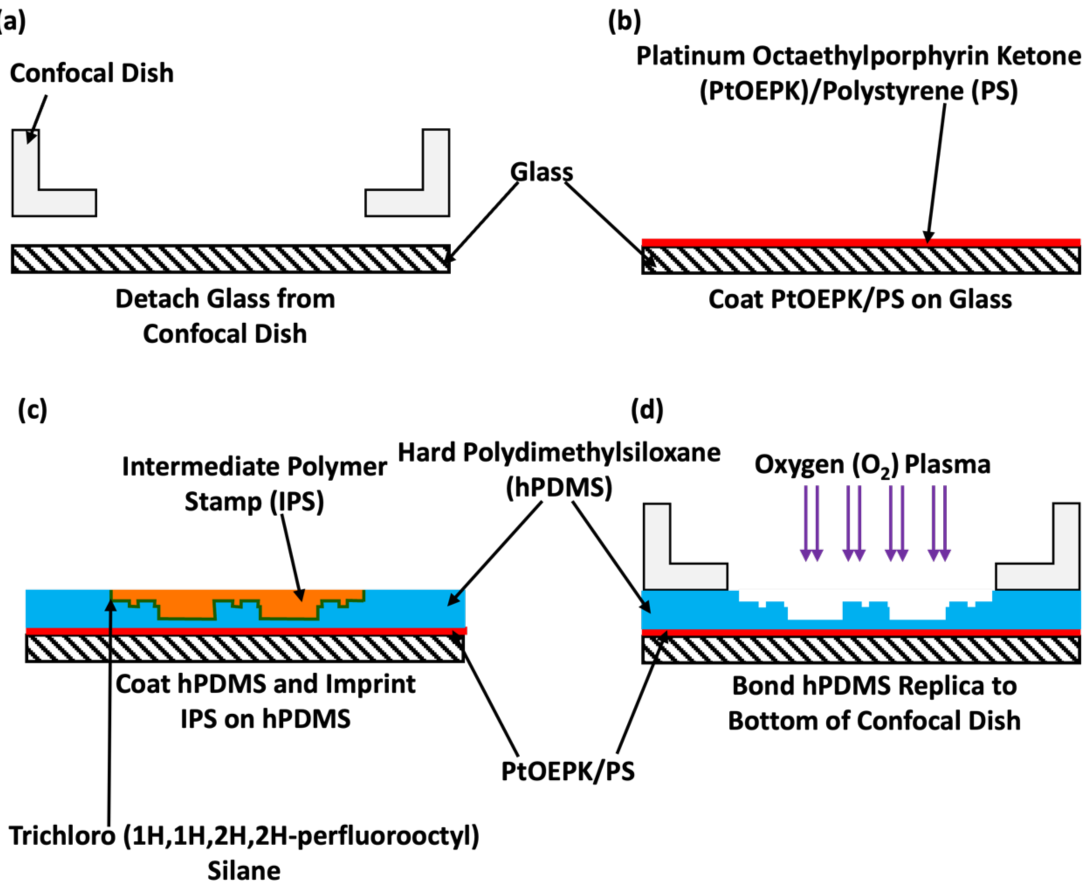


**Supplementary Figure S1.** Fabrication technology of pattern hard polydimethylsiloxane (hPDMS) guiding platform for oxygen ($O_2$) detection during cell migration. (a) Detachment of glass from confocal dish. (b) Coat platinum octaethylporphyrin ketone/polystyrene on glass. (c) Coat hPDMS on glass and imprint IPS on hPDMS. (d) Bond to confocal dish.

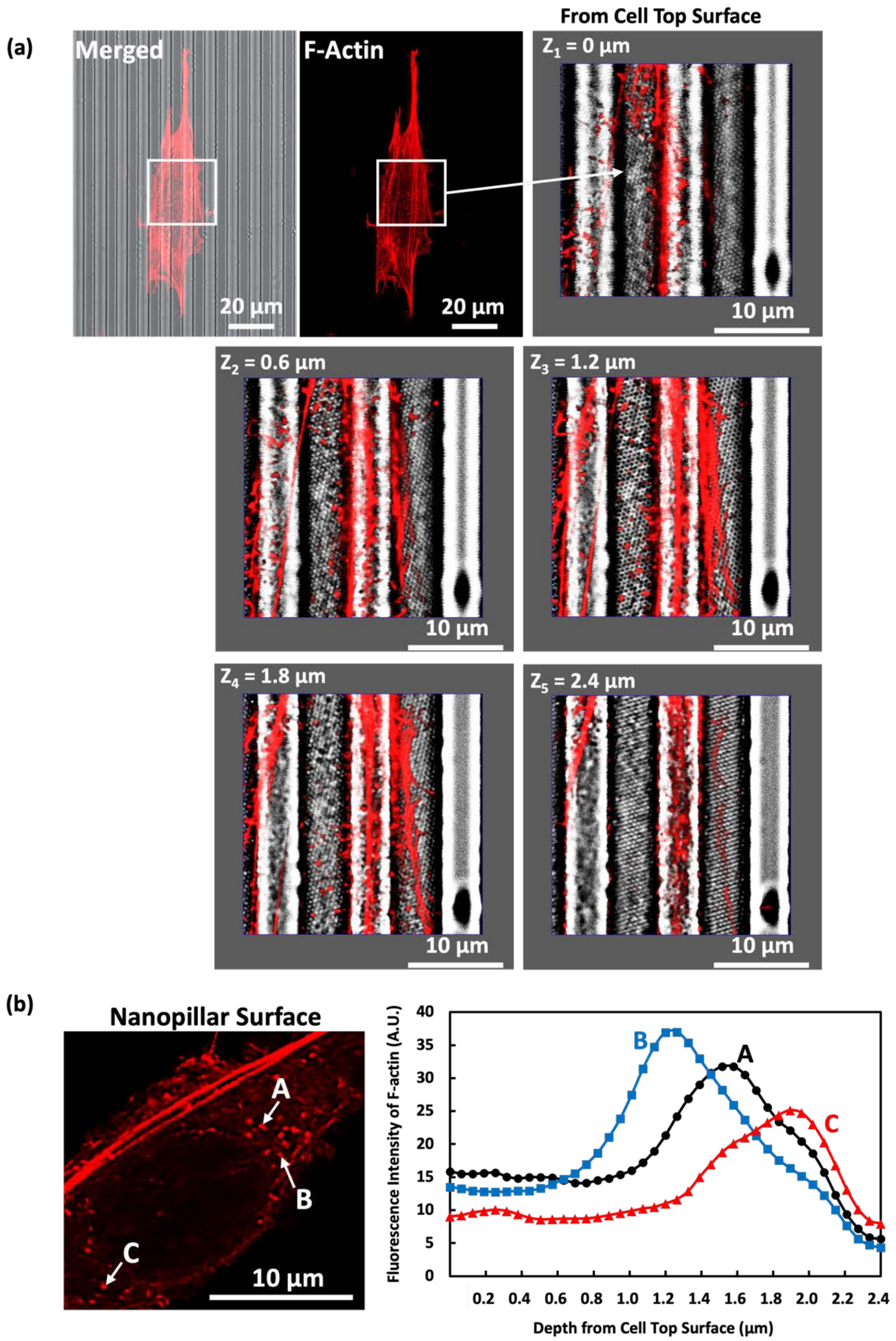


**Supplementary Figure S2.** Depth distribution of actin filaments (F-actin) of MC3T3-E1 cell on nanopillars. (a) Fluorescence images of F-actin from 0 to 2.4 μm below cell top surface. (b) Fluorescence intensity of three F-actin dots as function of depth from cell top surface.
